# Introduction of contraceptive implants as a family planning strategy in Honduras and Chiapas: Results from the Salud Mesoamerica Initiative (SMI)

**DOI:** 10.1371/journal.pone.0362270

**Published:** 2026-11-06

**Authors:** Adrián Santoro, Natalia Basualdo, Federico Rolón, Lucía Bartolomeu, Camila Volij, Matt Simon, Brittany Hagedorn, Diego Ríos-Zertuche, Santiago Esteban, Adolfo Rubinstein

**Affiliations:** 1 Center for Implementation and Innovation in Health Policies, Institute for Clinical Effectiveness and Health Policy, Buenos Aires, Argentina; 2 Primary Health Care, Global Development Division, Gates Foundation, Seattle, Washington, United States of America; 3 Institute for Disease Modeling, Global Health Division, Gates Foundation, Seattle, Washington, United States of America; 4 Health, Nutrition and Population Division, Inter-American Development Bank, Washington, District of Columbia, United States of America; 5 Social Protection and Health Division, Inter-American Development Bank, Washington, District of Columbia, United States of America; Institute of Public Health from Guanajuato State, MEXICO

## Abstract

Expanding access to a broad range of contraceptive methods is critical for reducing unintended pregnancies and improving reproductive health. This study evaluates the introduction of subdermal contraceptive implants in the selected areas of Honduras and Chiapas, Mexico, in the context of the Salud Mesoamérica Initiative, a results-based financing program focused on disadvantaged populations. Data were obtained from household surveys conducted between 2012 and 2022 among women aged 15–49 years in selected intervention and comparison municipalities in Chiapas, Mexico, and Honduras, using a two-stage sampling design. Indicators included contraceptive prevalence rates, reasons for method switching, and adoption by sociodemographic characteristics. Findings show a substantial increase in implant use in both settings over time. In the selected areas of Honduras, implant uptake increased gradually, reaching 7.9% in the 2022 SMI household survey, while in the selected areas of Chiapas use rose to 7.2%. Younger women aged 20–29 years and those with full literacy exhibited the highest adoption rates. In the selected areas of Chiapas and Honduras, implants most often replaced non-modern and short-acting hormonal methods, suggesting successful integration into the existing method mix. Variations in adoption across sociodemographic subgroups highlight persistent inequities. The results underscore the potential of subdermal implants to expand access to long-acting reversible contraception in resource-limited settings. To sustain and enhance this progress, strategies must address sociocultural and structural barriers, ensuring equitable access across all population groups and mitigating service disruptions. These findings may inform future strategies to expand informed contraceptive choice in similar resource-limited settings; their effects on reproductive health outcomes require further evaluation.

## Introduction

When it comes to family planning, variety matters, broadening family planning choices may lead to greater adoption and sustained use, effectively preventing unplanned pregnancies [1]. In the Mesoamerican region, high rates of unintended pregnancies highlight the need for more effective and accessible contraceptive methods, particularly for vulnerable populations. From 2015 to 2019, there was an average annual rate of 69 unintended pregnancies per 1000 women aged 15–49 in Latin America, compared to 35 in Europe and North America [2,3]. Despite the proven efficacy of subdermal implants as a long-term and reversible contraceptive option, their adoption remains limited in low and middle-income countries (LMICs). Access to these methods is often hindered by systemic barriers, including low healthcare infrastructure, cultural barriers, cost limitations, and a lack of awareness among providers and users [4]. Although the Food and Drug Administration (FDA) approved the etonogestrel implant (Implanon) in 2006, it has not been formally incorporated into the mix of contraceptive methods offered by Ministries of Health across Latin America compared to other regions [5]. However, good levels of adherence have been reported in settings where implants have been available to users [6].

The contraception method mix available and utilized within a population is frequently shaped by the policies and guidelines of healthcare providers. Historically, we’ve observed how this influence has impacted usage trends. For instance, in the 1980s, intrauterine devices (IUDs) were heavily promoted in several countries across Latin America and the Caribbean, leading to a high prevalence of their use. Subsequently, with the emergence of the HIV epidemic, the promotion of condoms significantly intensified due to their ability to prevent sexually transmitted diseases, thereby altering the dominant method mix. Similarly, the evolution of the contraceptive pill with lower hormone doses contributed to its growing acceptance in various settings [7].

Subdermal implants, a type of LARC (long-acting reversible contraceptives), are a highly effective and convenient method of contraception, offering long-term protection against unintended pregnancies [8]. These small, flexible rods are inserted under the skin of the upper arm, releasing a steady dose of progestin to prevent ovulation and thicken cervical mucus, thereby providing up to three to five years of reliable contraception.

One of their key advantages is reversibility—fertility typically returns quickly after removal, making them an excellent choice for individuals who may want children in the future. Subdermal implants are discreet, require minimal maintenance, and are not dependent on daily adherence, which enhances their effectiveness compared to user-dependent methods. This method mainly benefits individuals seeking a low-maintenance, reversible option that ensures long-term reproductive autonomy while retaining flexibility [8].

There is limited evidence on the use, acceptability, impact, and integration of subdermal implants into the contraceptive method mix in LMICs. Few studies have explored how these methods perform in resource-constrained settings, including their potential to address unmet needs for family planning and their integration into existing healthcare systems [7]. This knowledge gap restricts the ability of policymakers, and healthcare providers and practitioners to develop targeted strategies that promote equitable access to implants and address cultural, logistical, and financial barriers to their use.

Our research is based on the secondary analysis of the databases of the Salud Mesoamérica Initiative (SMI). SMI was a results-based financing program implemented across several countries in the region, aiming, among other goals, to increase family planning uptake. This context provides a valuable opportunity to study population acceptance of a new contraceptive method, such as the implant [9].

This study, aiming to describe the introduction of the contraceptive implant in the selected areas of Honduras and Chiapas, and its utilization among women within the context of the SMI, in order to provide critical insights into the potential of integrating a new LARC method in health systems that already offer a range of contraceptive options. Our research specifically focused on identifying the factors associated with implant adoption.

## Materials and methods

### Study design

This observational and exploratory study is a secondary analysis of repeated cross-sectional household surveys conducted through SMI in the selected areas of Chiapas and Honduras, including retrospectively reported monthly contraceptive use during the 24 months preceding each survey. The analysis included two repeated cross-sectional household survey rounds in the selected areas of Chiapas and three in the selected areas of Honduras.

Our analysis incorporated indicators of current contraceptive method usage derived from multiple-response questions. This approach allowed each participant to report the use of multiple contraceptive methods concurrently. Consequently, the percentage for each method reflects the proportion of respondents selecting that specific method.

This analysis used household survey data from the SMI baseline and second follow-up in the selected areas of Chiapas, Mexico (2012–2013 and 2018, respectively), and from the baseline, second follow-up, and third follow-up in the selected areas of Honduras (2013, 2017, and 2022, respectively). Household survey data were not available at the first follow-up for either setting. These labels refer to measurement rounds of the SMI evaluation, rather than to separate studies of contraceptive implants.

### Ethics statement

The study is based on a secondary data analysis generated by the Salud Mesoamerica Initiative from baseline and follow-up measurements. At the time of data collection written informed consent was obtained from all participants. The study received approval from the institutional review board (IRB) from the University of Washington, partnering data collection agencies, and the Ministry of Health in each country. For the study presented in this article, the authors accessed public and anonymized datasets on April 1st., 2024. The authors had no access to information that could identify individual participants.

### Setting

We used data from the household surveys. These surveys were conducted with women aged 15–49 years. Family planning questions were asked to women who declared their marital status as “married” or “partnered”. This analysis included household survey data from selected intervention and comparison municipalities in Chiapas, Mexico, and Honduras. In Honduras, the SMI baseline household survey included selected municipalities within the departments of Choluteca, Comayagua, Copán, Cortés, Intibucá, La Paz, Lempira, Olancho, and Valle. The findings refer to the selected study areas and should not be interpreted as estimates for the entire state of Chiapas or for Honduras as a whole [10,11].

### Participants

The study was conducted within the framework of the Salud Mesoamérica Initiative (SMI), a results-based financing program implemented between 2012 and 2022. This initiative targeted selected geographic areas to reach populations in the lowest wealth quintile, with this analysis focusing on selected municipalities in Chiapas, Mexico, and Honduras. Originally, follow-up assessments were planned at 18, 36, and 54 months after program launch, although these timelines were later modified. The data collection periods, as detailed above, spanned a decade, during which significant socio-economic changes occurred. Notably, the final follow-up survey was conducted amidst the COVID-19 pandemic, which may have influenced health behaviors and service access.

This analysis used repeated cross-sectional household surveys conducted in the selected intervention and comparison municipalities, comprising baseline and second follow-up data in Chiapas and baseline, second follow-up, and third follow-up data in Honduras.

The survey methodology has been explained in detail in previous SMI publications [9]. The surveys employed a two-stage random sampling method to collect data while ensuring the results accurately represent the overall population. Primary sampling units (PSUs) were randomly selected from a larger pool in the first stage. In the second stage, a sample of individuals or households was drawn from each selected primary survey unit. This approach balances cost-effectiveness with statistical precision.

### Outcomes

The outcomes, including contraceptive method use and reasons for switching/discontinuation, were measured through data collected during the household surveys. Current contraceptive method usage was assessed at the time of each survey through multiple-response questions. Additionally, monthly-based use of contraceptive methods and reasons for method switch or discontinuation were collected retrospectively for a reference period of two years prior to each survey.

The indicators related to current use are:

- Percentage of use of each method among eligible women in need of contraception.
  - Numerator: Number of women in need of contraceptives using a given method.
  - Denominator: Number of women in need of contraception.
- Percentage of use of modern methods - like permanent methods (sterilization), short-acting hormonal methods (pill, injectables and vaginal ring), long-acting reversible contraception (implants and IUDs), barrier methods (condoms, diaphragm, sponge), and emergency contraception - used among women in need of contraception.
  - Numerator: Number of women in need of contraception using a modern method.
  - Denominator: Number of women in need of contraception.

We also calculated monthly-based use of contraceptive methods and reasons for method switch or discontinuation in the household survey. This information was available for a reference period of two years before the survey. Data for this purpose were gathered from a specific section of the household survey. A trained interviewer prompted each woman to reconstruct, month by month, her use or discontinuation of contraceptive methods, recording the monthly information in a dedicated grid. The indicators of monthly use of contraceptive methods and implants were specifically calculated based on the following definitions:

- Percentage of monthly use of modern contraceptive methods
  - Numerator: number of married or partnered women who reported using any modern contraceptive method - like permanent methods (sterilization), short-acting hormonal methods (pill, injectables and vaginal ring), long-acting reversible contraception (implants and IUDs), barrier methods (condoms, diaphragm, sponge), and emergency contraception - in a given month of the reference period.
  - Denominator: number of married or partnered women who did not report a pregnancy, birth, or termination of method used for that month
- Percentage of monthly implant use
  - Numerator: number of married or partnered women who reported using contraceptive implants in a given month of the reference period.
  - Denominator: number of married or partnered women in need of contraception who have not reported a pregnancy, birth, or termination of the method used for that month
- Percentage of reasons to switch to implant:
  - Numerator: number of women who, having reported monthly contraceptive implant use for the first time in the reference period, declared switching for a given reason.
  - Denominator: number of married or partnered women who reported using an implant as a contraceptive method for the first time in the reference period.
- Percentage of women who reported a live birth in the last year stratified by age:
  - Numerator: number of women from specific age groups who reported a live birth in the last year.
  - Denominator: number of women from specific age groups

### Covariates

Primary stratification variables included country (Mexico -Chiapas-; Honduras) and measurement round (baseline and second follow-up in Chiapas; baseline, second follow-up, and third follow-up in Honduras), following the SMI evaluation nomenclature. Sociodemographic covariates collected through the household surveys, such as age and educational attainment were also used as stratification factors to characterize outcome distributions. Women were classified as fully literate if they reported being able to both read and write, and as partially literate if they reported being able to read but not write.

### Data sources

We used the pre-defined category “women in need of contraception” (those who are married or in a relationship as defined in the household surveys administered to women) as inclusion criteria. Women who reported no sexual activity, had never had sexual intercourse, were menopausal, were unable to conceive for reasons other than contraceptive sterilization, were pregnant, or wished to become pregnant at the time of the survey were excluded from the analysis.

### Bias

The extrapolation of findings from the analytical sample to the broader study population, and subsequently to the target population, relies on the assumption that the two-stage random sampling methodology employed by the SMI accurately represents the specified population (married/partnered women aged 15–49 in need of contraception within the selected study municipalities in Chiapas, Mexico, and Honduras). Potential sources of bias include response bias inherent in self-reported data on sensitive topics like contraceptive use, as well as potential recall bias for retrospective monthly usage. While efforts were made during survey design to minimize these, they remain inherent limitations.

### Data analysis

To assess implant usage as a contraceptive method, proportions were estimated based on self-reported use among women, encompassing both ‘current use’ and retrospectively reported monthly use. Proportions were reported with 95% confidence intervals. Within each survey round and selected study area, changes in each contraceptive-use category were estimated as the end-of-period minus start-of-period proportion over the 24-month retrospective reference period, restricting both estimates to women with observed data at both endpoints. Paired contrasts were calculated from jointly estimated survey-weighted proportions and their covariance, incorporating weights and clusters information. Differences were expressed in percentage points with t-based 95% confidence intervals; two-sided Wald t tests used the survey design degrees of freedom. P values were not adjusted for multiple comparisons, and the analyses were considered exploratory. Results are reported in S1 Table. The procedure is implemented in functions/table1Data.R and displayed by scripts/repo_table_01.R.

All statistical analyses were conducted using the R statistical software.

## Results

### Eligibility

As mentioned in the previous section, the use of contraceptive methods in the subgroup of women considered “in need of contraception” was described. Fig 1 shows how the analyzed sample was arrived at according to country and follow-up.

**Fig 1:**
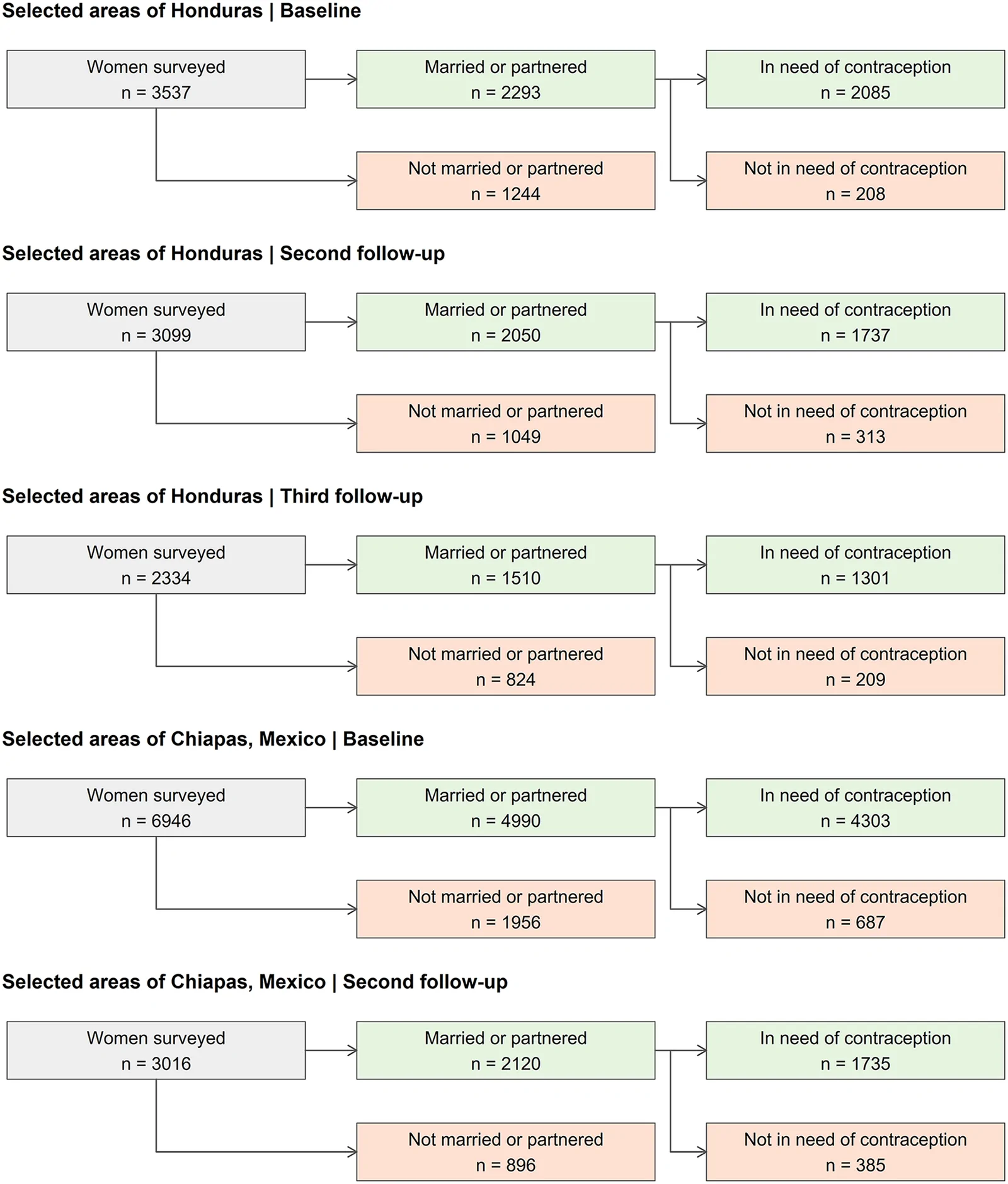
Flowchart of eligibility for “women in need of contraception”.

### Implants

Contraceptive implant use was assessed at baseline and the second follow-up in the selected areas of Chiapas, Mexico, and at baseline, the second follow-up, and the third follow-up in the selected areas of Honduras. Implant use increased between baseline and the latest available household survey in the selected areas of Chiapas and Honduras (Table 1 and Fig 2). Throughout the 24-month retrospective period prior to each follow-up, implant use increased progressively. During the second follow-up, adoption rates accelerated more rapidly in the selected areas of Chiapas than in the selected areas of Honduras. At the latest available household survey, current implant use was 7.9% (95% CI: 5.2–11.8) in the selected areas of Honduras and 7.2% (95% CI: 5.7–9.0) in the selected areas of Chiapas. These estimates derive from the current contraceptive use question and differ from the retrospective monthly estimates presented in Table 1 and Fig 2, as the questions did not apply to exactly the same population. Paired comparisons of the endpoints of the retrospective period showed increases in implant use at the latest available survey in the selected areas of Honduras (3.36 percentage points, 95% CI: 1.02–5.70; t = 2.87, df = 66, p = 0.006) and Chiapas (2.48 percentage points, 95% CI: 1.44–3.52; t = 4.75, df = 80, p < 0.001). These comparisons concern retrospective monthly use, not the current-use estimates above (S1 Table). This level does not appear to represent a ceiling, as the upward trend suggests continued growth in future measurements. The consistent upward trajectory indicates sustained acceptance of this contraceptive method across both regions, despite their different baseline starting points.

**Table 1:** Percentage of use of contraceptive methods by country and follow-up, at start and end of 24-month period prior to the survey.

Follow-up	Method	Honduras								Chiapas							
		Start				Finish				Start				Finish			
		n	Percentage	Lower bound	Upper bound	n	Percentage	Lower bound	Upper bound	n	Percentage	Lower bound	Upper bound	n	Percentage	Lower bound	Upper bound
Baseline	No method	1,074	49.42	44.96	53.88	630	34.14	29.62	38.66	2,399	57.33	53.72	60.94	1,759	44.52	40.92	48.11
	Non-implant modern methods	897	45.10	40.85	49.35	1,302	58.58	54.04	63.12	1,278	37.58	34.06	41.11	1,785	46.97	43.46	50.47
	Implant	1	0.12	0.00	0.37	2	0.08	0.00	0.18	49	1.25	0.71	1.78	96	2.09	1.28	2.91
	Non-modern methods	91	4.62	3.14	6.09	132	6.56	4.55	8.56	146	3.22	2.53	3.91	275	5.78	4.74	6.83
	No response	11	0.74	0.10	1.38	8	0.65	0.04	1.27	19	0.62	0.12	1.12	15	0.64	0.08	1.21
2nd. Follow-up	No method	750	38.11	33.17	43.04	354	22.15	18.03	26.27	1,006	55.26	49.31	61.21	755	45.17	39.07	51.26
	Non-implant modern methods	852	57.96	52.53	63.39	1,200	71.88	67.19	76.57	533	37.71	32.37	43.05	699	44.40	38.79	50.02
	Implant	29	1.77	0.57	2.97	65	3.41	1.84	4.98	106	4.31	3.00	5.62	179	6.79	5.40	8.17
	Non-modern methods	39	1.61	0.84	2.37	55	2.49	1.57	3.41	24	1.05	0.51	1.59	38	1.96	1.01	2.91
	No response	3	0.56	0.00	1.47	1	0.07	0.00	0.20	23	1.67	0.54	2.80	22	1.68	0.56	2.81
3rd. follow-up	No method	344	32.62	27.06	38.18	163	20.28	14.18	26.38	–	–	–	–	–	–	–	–
	Non-implant modern methods	552	52.18	46.29	58.07	687	61.47	55.84	67.10	–	–	–	–	–	–	–	–
	Implant	46	3.53	1.98	5.08	100	6.90	4.26	9.53	–	–	–	–	–	–	–	–
	Non-modern methods	76	9.04	5.70	12.39	69	8.41	4.98	11.84	–	–	–	–	–	–	–	–
	No response	35	2.63	1.08	4.17	32	2.94	1.21	4.68	–	–	–	–	–	–	–	–

**Fig 2:**
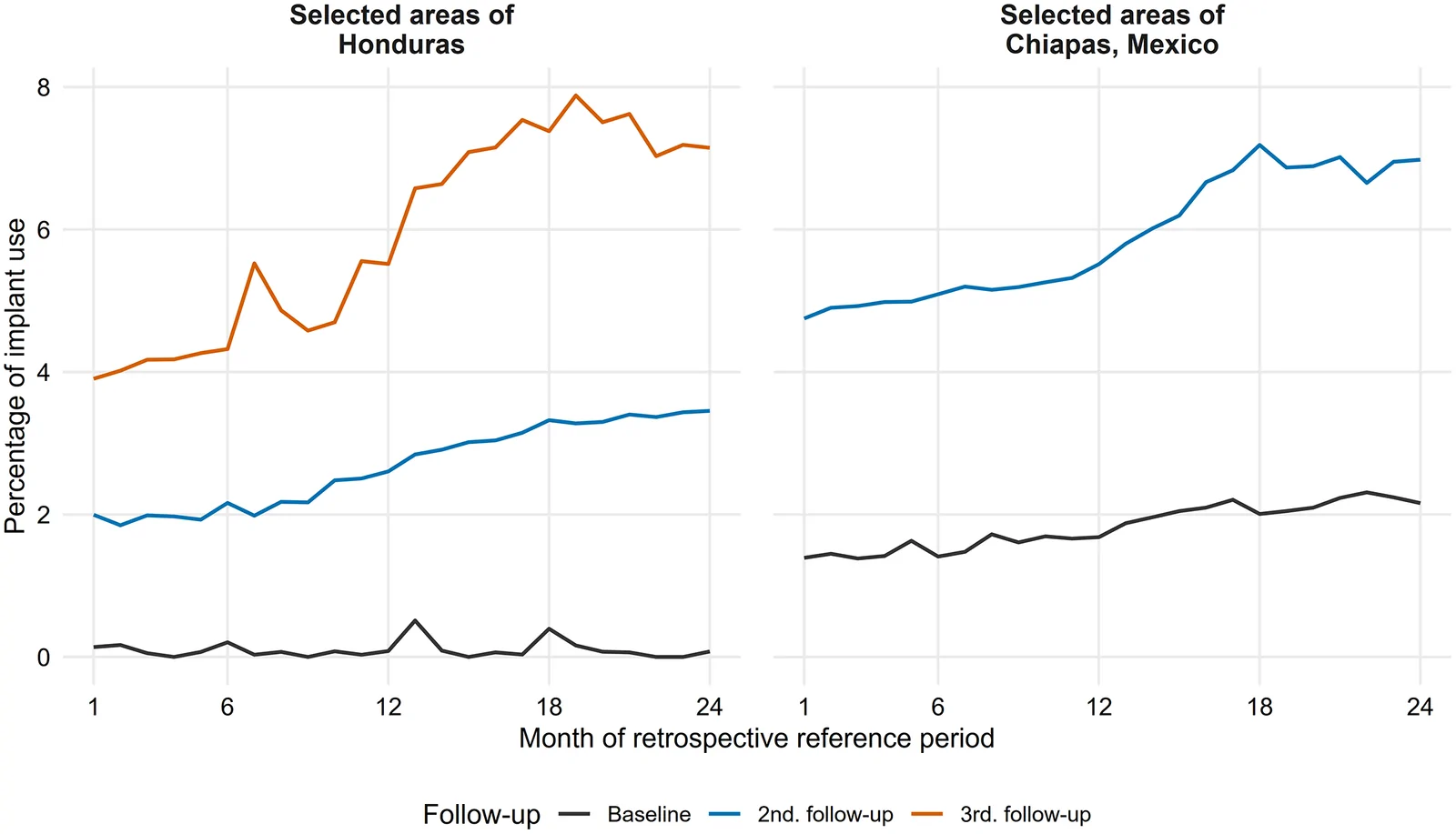
Percentage of implant use as a contraceptive method in the 24 months prior to the survey, by month and country.

Comparative results for Honduras and Chiapas provide further depth to these observations. Although data for Chiapas were more limited, with follow-ups available only at baseline and the second follow-up, significant patterns emerged. Implant use was initially more frequent in the selected areas of Chiapas than in the selected areas of Honduras. Table 1 shows changes in the contraceptive method mix that varied by setting and survey round. These included changes in implant use, use of other methods, non-use, and non-response; the patterns do not indicate a uniform decline in all non-implant categories. Within the 24-month retrospective periods, non-modern method use increased in the selected areas of Honduras at baseline (1.94 percentage points, 95% CI: 0.62–3.26; p = 0.004) and the second follow-up (0.89 percentage points, 95% CI: 0.37–1.40; p < 0.001), and in the selected areas of Chiapas at baseline (2.48 percentage points, 95% CI: 1.75–3.22; p < 0.001) and the second follow-up (0.91 percentage points, 95% CI: 0.23–1.60; p = 0.010). At the third follow-up in the selected areas of Honduras, the estimated change was −0.66 percentage points (95% CI: −2.71 to 1.39; p = 0.522), providing no clear evidence of a change. These are comparisons within survey rounds, not between rounds (S1 Table).

Within each survey round, retrospectively reported contraceptive histories indicated that implant initiation was commonly preceded by injectable use or non-use of contraception. These transitions were reconstructed within individual women’s reported histories, rather than by following the same women across survey rounds. A notable percentage of implant adoption also occurred as a post-birth family planning method, further emphasizing its role in the overall shift toward modern contraceptive methods. Fig 3 describes the methods used, or non-use of contraception, before implant initiation; it does not depict trends in implant prevalence or in the population prevalence of other contraceptive methods. An aggregate estimate obtained by summing the survey-design-adjusted percentages for modern contraceptive methods, excluding traditional methods such as calendar-based methods, indicated that modern method use in the selected areas of Honduras was approximately 50% at the end of the 24-month reference period preceding baseline and 75% at the end of the corresponding period preceding the third follow-up. These aggregate estimates are not displayed separately in the tables or figures. It is important to note that one non-modern method (the rhythm method) is mentioned as a method replaced by approximately 10% of women. However, the replacement of injectables and non-use of contraceptives whatsoever are still the most frequent categories, with about half of the replacements. Interestingly, Chiapas exhibited a higher proportion of implants replacing the non-use of contraceptives. (Fig 3).

**Fig 3:**
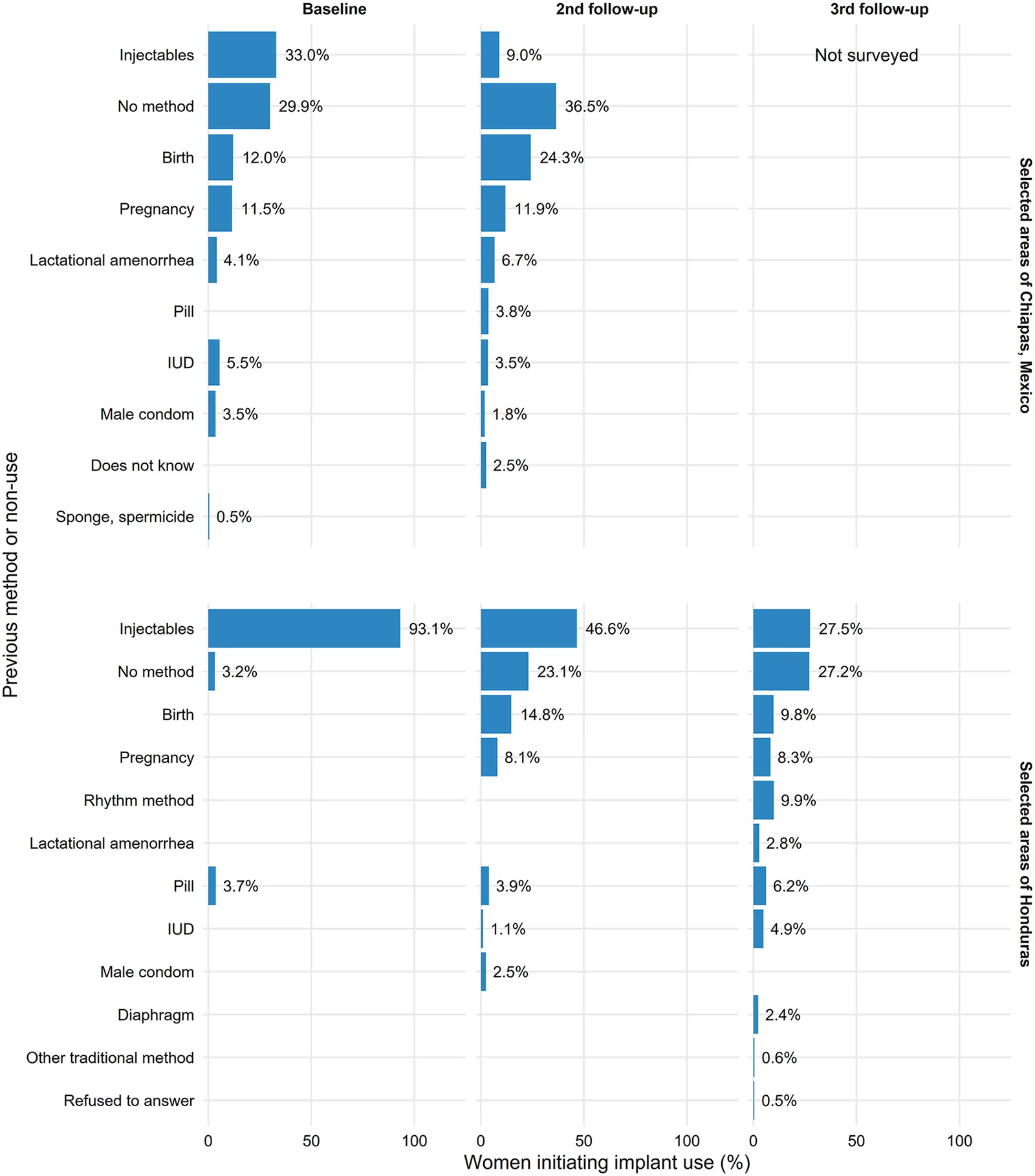
Replacement of contraceptives by Implants at each follow-up, and by country.

### Contraceptive uptake by sociodemographic characteristics

Looking at the post-baseline follow-ups, where the implant appears as a method of increasing use in both selected study areas, women with full literacy generally show higher adoption rates. While in the selected areas of Honduras both non-literate (8.7%, CI: 2.1–29.5) and fully literate (9.0%, CI: 6.7–12.0) women show similar implant uptake, in the selected areas of Chiapas implant uptake was higher for fully literate women: 8.0% (CI: 6.4–10.0) at 2nd. follow-up, compared to non-literate women (3.4%, CI: 2.1–5.5).

Implant use was generally higher among younger women in the selected areas of Chiapas and Honduras. By third follow-up in the selected areas of Honduras, younger age groups showed higher adoption rates (15–19 years: 19.2%, 20–29 years: 15.6%) compared to older groups (30–39 years: 5.6%, 40–49 years: 2.4%). In the selected areas of Chiapas, similar age-related trends emerged, though less pronounced, with the 20–29 age group showing the highest adoption rates at 2nd. follow-up (12.9%), followed by 15–19 years (7.2%). In the selected areas of Chiapas, implant use at the second follow-up was 4.6% among women aged 30–39 years and 1.1% among those aged 40–49 years (Table 2). In the selected areas of Honduras, there was an increase in implant usage among women aged 15–19 years. Chiapas shows a similar trend, though the increase is more moderate, with the 20–29 age group showing the highest adoption rates.

**Table 2:** Implant use by socioeconomic characteristics, follow-up and country.

Follow-up	Variable		Honduras				Chiapas		
		n	Percentage	Confidence interval		n	Percentage	Confidence interval	
				Lower bound	Upper bound			Lower bound	Upper bound
2nd. Follow-up	Age group								
	15-19 years	8	3.7	1.4	9.5	14	7.2	3.8	13.2
	20-29 years	34	5.5	2.9	10.1	116	12.9	9.9	16.7
	30-39 years	26	3.3	1.9	5.6	41	4.6	3.3	6.4
	40-49 years	3	0.4	0.1	1.4	7	1.1	0.5	2.4
	Literacy level								
	No	8	2.6	0.8	8.0	15	3.4	2.1	5.5
	Partial	5	2.0	0.8	5.3	25	5.6	3.6	8.5
	Full	58	3.8	2.3	6.2	138	8.0	6.4	10.0
	Marital status								
	Married	16	2.2	0.9	5.2	65	4.9	3.6	6.6
	Partnered	55	4.3	2.9	6.3	114	8.3	6.4	10.7
3rd. Follow-up	Age group								
	15-19 years	17	19.2	10.6	32.1				
	20-29 years	89	15.6	11.4	21.1				
	30-39 years	36	5.6	3.7	8.4				
	40-49 years	7	2.4	0.6	9.7				
	Literacy level								
	No	9	8.7	2.1	29.5				
	Partial	17	5.3	2.9	9.5				
	Full	122	9.0	6.7	12.0				
	Marital status								
	Married	26	5.3	2.6	10.7				
	Partnered	123	10.2	7.7	13.6				

Both selected study areas show higher adoption rates among partnered women compared to married women across all time points, with the difference becoming more pronounced over time. By 3rd. follow-up in the selected areas of Honduras, partnered women showed 10.2% adoption compared to 5.3% among married women. In the selected areas of Chiapas, at the second follow-up, partnered women showed 8.3% adoption versus 4.9% among married women (Table 2).

### Analysis of pooled data

The analysis of contraceptive implant adoption across the selected areas of Honduras and Chiapas shows significant temporal and sociodemographic patterns. The data demonstrate a substantial increase in implant utilization from baseline to 36-month follow-up across both selected study areas (p < 0.05), with Chiapas exhibiting consistently higher adoption rates throughout the study period. At baseline, implant prevalence in the selected areas of Chiapas (2.32%, CI: 1.60–3.36) was markedly higher than in the selected areas of Honduras (0.37%, CI: 0.11–1.23). This disparity persisted at the 36-month follow-up, with Chiapas reaching 6.7% (CI: 5.45–8.22) compared to Honduras at 3.44% (CI: 2.23–5.28).

Examination of age-stratified data indicates notable age-dependent adoption patterns. Young women aged 20–29 demonstrated the highest uptake, with utilization increasing from 3.76% (CI: 2.54–5.53) at baseline to 11.3% (CI: 8.91–14.23) at 36 months (p < 0.05). This finding suggests particular receptivity to implant adoption among women in their peak reproductive years. Conversely, women aged 40–49 showed consistently lower adoption rates, as suspected, increasing modestly from 0.15% (CI: 0.02–1.09) to 1.0% (CI: 0.49–2.04) but statistically significant (p < 0.05).

Literacy levels also demonstrated a pattern of association with implant adoption. Women with full literacy showed higher utilization rates, increasing from 2.75% (CI: 1.91–3.94) to 7.01% (CI: 5.76–8.51) at 36 months (p < 0.05). However, it is noteworthy that women with no literacy also exhibited increased adoption over time (p < 0.05), rising from 0.86% (CI: 0.42–1.76) to 3.30% (CI: 2.14–5.04), indicating successful program reach across literacy levels (Table 3).

**Table 3:** Implant use by socioeconomic characteristics, follow-up. Pooled data.

Variable		Baseline			2nd. Follow-up		
		Percentage	Confidence interval		Percentage	Confidence interval	
			Lower bound	Upper bound		Lower bound	Upper bound
Area	Honduras	0.37	0.11	1.23	3.44	2.23	5.28
	Chiapas	2.32	1.60	3.36	6.7	5.45	8.22
Age group	15-19 years	2.23	0.88	5.44	6.35	3.74	10.58
	20-29 years	3.76	2.54	5.53	11.3	8.91	14.23
	30-39 years	1.44	0.84	2.44	4.3	3.23	5.71
	40-49 years	0.15	0.02	1.09	1.0	0.49	2.04
Literacy level	No	0.86	0.42	1.76	3.30	2.14	5.04
	Partial	1.41	0.79	2.49	5.05	3.39	7.46
	Full	2.75	1.91	3.94	7.01	5.76	8.51
Marital status	Married	1.47	0.90	2.39	4.38	3.31	5.78
	Partnered	2.47	1.60	3.79	7.38	5.92	9.16

## Discussion

Our findings indicate an increase in contraceptive implant use in the selected areas of Chiapas and Honduras, with variation across sociodemographic groups. However, adoption patterns varied across the selected study areas and socio-demographic groups, highlighting the need for multilevel, context-specific strategies to ensure equitable access to and uptake of implants.

To address missed opportunities in contraceptive provision and expand contraceptive choice through access to subdermal implants, these devices were introduced as part of national sexual and reproductive health policies in Honduras and Chiapas. Although we hypothesized that intervention municipalities could have been more successful in promoting adoption than comparison municipalities, this was not the case, and thus, we further analyzed intervention and comparison municipalities together. Implant use increased over the observation period in both study groups; this analysis cannot determine the contribution of program implementation to those changes. While both areas experienced growth in implant use, Chiapas consistently exhibited higher adoption rates than Honduras throughout the study period. At baseline, the implant utilization rate in Chiapas was 2.32%, increasing to 6.7% at the 36-month follow-up. These findings align with trends reported by Darney et al. during the same period, which showed an increase in implant use from 1.1% to 4.5% between 2009 and 2018 (p < 0.001), with the most significant rise occurring among adolescents (from 2.5% to 12.2%; p < 0.001) [12]. The difference in implant utilization rates between the two settings may be attributed to several factors, including pre-existing levels of awareness and acceptance of implants, variations in healthcare infrastructure and service delivery models, and cultural norms and preferences related to family planning.

Data published following the baseline analysis of the Salud Mesoamérica Initiative identified the need to improve services for individuals of indigenous ethnicity, those with low educational attainment, people living in extreme poverty, the uninsured, and adolescents [13]. Our findings further emphasize the need to consider sociodemographic factors when designing and implementing family planning programs. Age emerged as a key predictor of implant adoption, with younger women, particularly those aged 20–29, demonstrating the highest uptake. The increase observed among adolescents in the selected areas of Honduras warrants further investigation into the factors associated with implant use in this age group. This highlights the need for targeted interventions that address the specific needs and preferences of young women, including comprehensive sexual and reproductive health education and youth-friendly services, and home sexual violence prevention, as it was noted as barriers in previous SMI publications [14].

Educational attainment and literacy levels also played a role in implant adoption, with women with higher education and literacy demonstrating higher utilization rates. This finding is consistent with the broader literature on contraceptive use, which often shows a positive correlation between education and adoption of modern methods [15]. Interestingly, the rate of adoption was observed to be higher among partnered women compared to married women. The reasons for the observed difference between partnered and married women were not assessed in this analysis. Future research should explore reproductive preferences and relationship dynamics without assuming that marital status reflects relationship stability or specific contraceptive motivations. Future research should also evaluate educational approaches that provide balanced information about contraceptive options, benefits, and risks, and explore voluntary male involvement in family planning while safeguarding women’s autonomy and contraceptive decision-making. Implant use also increased over time among women reporting no literacy in the selected areas of Chiapas and Honduras. These descriptive findings do not establish the effectiveness of SMI communication strategies or effects on unintended pregnancies.

Future implementation research should evaluate strategies to improve access to implants among younger women, including counseling that supports informed choice, affordability, and timely access to insertion and removal services.

Evidence from a pay-for-performance scheme for LARC counseling provides relevant context for results-based financing, but does not establish an implant-specific effect or explain the changes observed in our study [16]. The sustainability of implant services beyond external program support was not assessed in this analysis. Future research should examine how financing, procurement, and service capacity affect continued access to contraceptive choice, including implant insertion and removal, in the selected study areas.

The observed fluctuations in implant use in the intervention areas of Honduras, particularly the slowdown during the third follow-up, warrant further investigation. While the COVID-19 pandemic is a potential contributing factor, as it disrupted healthcare services globally, other factors such as supply chain issues, changes in service delivery models, or shifts in community perceptions of implants could also have played a role. Further research is needed to understand the specific reasons for these fluctuations and to develop strategies to mitigate similar disruptions in the future.

The introduction of contraceptive implants presents an opportunity to expand contraceptive choice in Latin America. Implementation strategies should support equitable access, informed choice, and continuity of implant use among women who wish to continue using the method.

In the specific context of Latin America, where access to healthcare and family planning services may be constrained, implants offer a practical solution. It is crucial, however, to acknowledge that successful implementation needs to address sociocultural factors that may influence implant acceptance [14]. This includes ensuring equitable access, affordability, and comprehensive healthcare provider training in insertion and removal procedures. In the selected study areas, culturally appropriate counseling should respect linguistic diversity, individual preferences, and reproductive autonomy, without assuming that Indigenous identity implies opposition to contraception.

Our study has some limitations. The observational nature of the study design does not allow us to establish causal relationships between the SMI intervention and the observed changes in implant utilization. The reliance on self-reported data may also introduce some recall and acquiescence bias [17]. Additionally, the data for Chiapas were limited to the baseline and second follow-up, which restricted our ability to make direct comparisons with Honduras at later time points. The restriction of the analysis to women who declared their marital status as “married” or “partnered” also limits the generalizability of the findings to unmarried women. Furthermore, data quality issues, particularly regarding the reasons for method switching in the selected areas of Chiapas, hampered some aspects of the analysis. The quality of the available data on reasons for switching from implants to other methods or discontinuing implant use was insufficient to support a reliable analysis. This limited our ability to assess barriers to continued implant use, including discontinuation related to menstrual changes or other adverse effects. Future studies should collect reliable data on these outcomes to guide strategies that support informed contraceptive choice, implant acceptance, and continuation among women who wish to continue using the method. The timing of implant introduction and expansion in the selected areas of Chiapas and Honduras could not be precisely established. Although qualitative evidence provides context for the selected areas of Chiapas, it does not quantify implant use before SMI [14]. Similarly, the available evidence does not establish the extent of implant use before SMI in the selected areas of Honduras. Consequently, the interval between local implementation and the surveys could not be reliably determined. These findings describe contraceptive implant use during the survey periods and should not be interpreted as estimates of current use in the selected areas of Chiapas and Honduras. This analysis did not assess service-level factors such as implant availability, stock-outs, or provider capacity. Future research linking household and health facility data could help clarify how these factors influence implant access and use in the selected areas of Chiapas and Honduras. Despite these limitations, our study provides valuable insights into the trends and patterns of contraceptive implant utilization in the selected areas of Honduras and Chiapas within the context of the SMI.

Our findings highlight the complex interplay between sociodemographic factors and contraceptive implant adoption. Differences across sociodemographic groups persisted alongside increases in implant use over the study period. The consistently higher rates in the selected areas of Chiapas may reflect differences in healthcare infrastructure, cultural factors, or implementation strategies between the selected study areas in Chiapas and Honduras. The strong age-related gradient, with higher adoption rates among younger women, highlights the need to further investigate older age groups, focusing on the reasons behind the adoption or non-adoption of contraceptive implants. One possible explanation for lower implant use among older women in the selected areas of Chiapas and Honduras is greater use of permanent contraceptive methods. However, this explanation was not evaluated in the present analysis and should be investigated in future studies. This suggests that targeted interventions for older women could be beneficial. Similarly, the persistent educational and literacy gradients indicate potential areas for focused outreach to reduce disparities in access and uptake. Future research should examine whether differences in implant use reflect contraceptive preferences, use of alternative methods, or barriers to access, particularly among groups facing socioeconomic disadvantage, to inform strategies that support equitable access and informed contraceptive choice. These findings can guide future prospective and qualitative studies involving women directly to better understand contraceptive preferences, experiences of implant use and discontinuation, and barriers to accessing services in the selected areas of Chiapas and Honduras.

Further research is necessary to explore these dynamics more deeply, particularly the motivations and barriers influencing contraceptive adoption and the broader implications for fertility trends along the follow-ups [14]. Such insights will be essential for designing policies and programs that effectively meet the diverse needs of women in these contexts.

## Conclusion

These findings underscore the potential of implants to address unmet needs for family planning and reduce unintended pregnancies in LMICs and highlight the importance of context-specific strategies for promoting equitable access and uptake. Future research should focus on exploring the factors that influence sustained adoption of implants, evaluating the impact of integrated interventions that address broader social determinants of reproductive health, and developing strategies to mitigate disruptions in service delivery, such as those caused by pandemics or other crises. These findings have important implications for future program implementation and policy development, suggesting the need for targeted strategies to address persistent disparities while maintaining the overall positive trajectory of implant adoption.

## Supporting information

S1 Table Paired changes in contraceptive method use over the 24-month retrospective period in the selected areas of Chiapas, Mexico, and Honduras. Survey-weighted paired differences, 95% confidence intervals and Wald t tests by category and survey round.
(DOCX)

## References

[1] Bertrand JT, Ross J, Sullivan TM, Hardee K, Shelton JD. Contraceptive method mix: Updates and implications. Glob Health Sci Pract. 2020;8(4):666–79. doi: 10.9745/GHSP-D-20-00229 33361234 PMC7784075

[2] Bearak J, Popinchalk A, Ganatra B, Moller AB, Tunçalp Ö, Beavin C, et al. Unintended pregnancy and abortion by income, region, and the legal status of abortion: Estimates from a comprehensive model for 1990-2019. Lancet Glob Health. 2020;8(9):e1152–61. 10.1016/S2214-109X(20)30315-6 32710833

[3] Bearak JM, Popinchalk A, Beavin C, Ganatra B, Moller AB, Tunçalp Ö. Country-specific estimates of unintended pregnancy and abortion incidence: A global comparative analysis of levels in 2015-2019. BMJ Global Health. 2022;7(3):e007151. doi: 10.1136/bmjgh-2021-007151 PMC8943721 35332057

[4] Houghton N, Báscolo E, Jara L, Cuellar C, Coitiño A, Del Riego A, et al. Rev Panam Salud Publica. 2022;46:e94. 10.26633/RPSP.2022.94 PMC9299390 35875315

[5] UNFPA LAC [Internet]. Estrategias de salud territorial para promover la utilización de dispositivos intrauterinos en América Latina y el Caribe. 2021. Available from: https://lac.unfpa.org/es/publications/estrategias-de-salud-territorial-para-promover-la-utilizaci%C3%B3n-de-dispositivos

[6] Maceira D, Oizerovich S, Perrotta G, de León RGP, Karolinski A, Suarez N, et al. Acceptability and continuation of use of the subdermal contraceptive implant among adolescents and young women in Argentina: A retrospective cohort study. Sex Reprod Health Matters. 2023;31(1):2189507. doi: 10.1080/26410397.2023.2189507 37042700 PMC10101666

[7] Ponce de Leon RG, Ewerling F, Serruya SJ, Silveira MF, Sanhueza A, Moazzam A, et al. Contraceptive use in Latin America and the Caribbean with a focus on long-acting reversible contraceptives: Prevalence and inequalities in 23 countries. Lancet Glob Health. 2019;7(2):e227–35. doi: 10.1016/S2214-109X(18)30481-9 30683240 PMC6367565

[8] Bahamondes L, Fernandes A, Monteiro I, Bahamondes MV. Long-acting reversible contraceptive (LARCs) methods. Best Pract Res Clin Obstet Gynaecol. 2020;66:28–40. 10.1016/j.bpobgyn.2019.12.002 32014434

[9] Mokdad AH, Colson KE, Zúñiga-Brenes P, Ríos-Zertuche D, Palmisano EB, Alfaro-Porras E, et al. Salud Mesoamérica 2015 Initiative: Design, implementation, and baseline findings. Popul Health Metr. 2015;13(1):3. doi: 10.1186/s12963-015-0034-4 25685074 PMC4327957

[10] Colson KE, Gagnier MC, Hernandez B, Mokdad AH. Honduras Baseline Household Census and Survey Data Quality Report [Internet]. 2013. Available from: https://www.saludmesoamerica.org/sites/default/files/toolkits/documents/Honduras%20Baseline%20Household%20Data%20Quality%20Report%20SMI.pdf

[11] SMI-Mexico Household Census and Survey Data Quality Report Second Follow-up Measurement [Internet]. Institute for Health Metrics and Evaluation. 2018. Available from: https://www.saludmesoamerica.org/sites/default/files/toolkits/documents/Chiapas%202nd%20Operation%20Household%20Survey%20Data%20Quality%20Report%20SMI.pdf

[12] Darney BG, Fuentes-Rivera E, Corbin A, Saavedra-Avendano B, Schiavon R. Trends in subdermal contraceptive implant use in Mexico 2009-2018: A population-based study. Int J Gynaecol Obstet. 2022;156(2):284–91. 10.1002/ijgo.13744 33999446

[13] Rios-Zertuche D, Blanco LC, Zúñiga-Brenes P, Palmisano EB, Colombara DV, Mokdad AH, et al. Contraceptive knowledge and use among women living in the poorest areas of five Mesoamerican countries. Contraception. 2017;95(6):549–57. doi: 10.1016/j.contraception.2017.01.005 28126542 PMC5493184

[14] Dansereau E, Schaefer A, Hernández B, Nelson J, Palmisano E, Ríos-Zertuche D, et al. Perceptions of and barriers to family planning services in the poorest regions of Chiapas, Mexico: A qualitative study of men, women, and adolescents. Reprod Health. 2017;14(1):129. doi: 10.1186/s12978-017-0392-4 29041977 PMC5646150

[15] Pazol K, Zapata LB, Tregear SJ, Mautone-Smith N, Gavin LE. Impact of contraceptive education on contraceptive knowledge and decision making: A systematic review. Am J Prev Med. 2015;49(2 Suppl 1):S46–56. doi: 10.1016/j.amepre.2015.03.031 26190846 PMC4532374

[16] Ma R, Cecil E, Bottle A, French R, Saxena S. Impact of a pay-for-performance scheme for long-acting reversible contraceptive (LARC) advice on contraceptive uptake and abortion in British primary care: An interrupted time series study. PLoS Med. 2020;17(9):e1003333. doi: 10.1371/journal.pmed.1003333 32925909 PMC7489538

[17] Hameed W, Ishaque M, Gul X, Siddiqui J-U-R, Hussain S, Hussain W, et al. Does courtesy bias affect how clients report on objective and subjective measures of family planning service quality? A comparison between facility- and home-based interviews. Open Access J Contracept. 2018;9:33–43. doi: 10.2147/OAJC.S153443 29760573 PMC5937485

