# Supplementary material for: Introduction of contraceptive implants as a family planning strategy in Honduras and Chiapas: Results from the Salud Mesoamerica Initiative (SMI)
Source: PLoS One. 2026 Nov 6;21(11):e0362270. doi: 10.1371/journal.pone.0362270 (PMC13742971; doi:10.1371/journal.pone.0362270)
Supplement: Supplementary file 1 [file pone.0362270.s001.docx]

S1 Table Paired changes in contraceptive method use

| **Area** | **Follow-up** | **Category** | **n paired** | **Start %** | **End %** | **Difference (pp)** | **95% CI (pp)** | **t** | **df** | **p** |
| --- | --- | --- | --- | --- | --- | --- | --- | --- | --- | --- |
| Selected areas of Honduras | Baseline (2013) | No method | 2069 | 49.51 | 34.18 | -15.33 | -17.72 to -12.93 | -12.71 | 98 | <0.001 |
| Selected areas of Honduras | Baseline (2013) | Non-implant modern methods | 2069 | 45.05 | 58.57 | 13.53 | 11.36 to 15.70 | 12.36 | 98 | <0.001 |
| Selected areas of Honduras | Baseline (2013) | Implant | 2069 | 0.12 | 0.08 | -0.05 | -0.32 to 0.22 | -0.36 | 98 | 0.718 |
| Selected areas of Honduras | Baseline (2013) | Non-modern methods | 2069 | 4.58 | 6.52 | 1.94 | 0.62 to 3.26 | 2.92 | 98 | 0.004 |
| Selected areas of Honduras | Baseline (2013) | No response | 2069 | 0.74 | 0.65 | -0.09 | -0.19 to 0.01 | -1.74 | 98 | 0.085 |
| Selected areas of Honduras | Second follow-up (2017) | No method | 1671 | 38.10 | 22.02 | -16.08 | -19.65 to -12.50 | -8.95 | 80 | <0.001 |
| Selected areas of Honduras | Second follow-up (2017) | Non-implant modern methods | 1671 | 57.97 | 71.99 | 14.03 | 10.42 to 17.64 | 7.74 | 80 | <0.001 |
| Selected areas of Honduras | Second follow-up (2017) | Implant | 1671 | 1.77 | 3.42 | 1.65 | 0.89 to 2.41 | 4.30 | 80 | <0.001 |
| Selected areas of Honduras | Second follow-up (2017) | Non-modern methods | 1671 | 1.61 | 2.49 | 0.89 | 0.37 to 1.40 | 3.44 | 80 | <0.001 |
| Selected areas of Honduras | Second follow-up (2017) | No response | 1671 | 0.56 | 0.07 | -0.49 | -1.39 to 0.42 | -1.07 | 80 | 0.286 |
| Selected areas of Honduras | Third follow-up (2022) | No method | 1051 | 32.42 | 20.28 | -12.14 | -15.90 to -8.39 | -6.45 | 66 | <0.001 |
| Selected areas of Honduras | Third follow-up (2022) | Non-implant modern methods | 1051 | 52.33 | 61.47 | 9.14 | 5.12 to 13.16 | 4.54 | 66 | <0.001 |
| Selected areas of Honduras | Third follow-up (2022) | Implant | 1051 | 3.54 | 6.90 | 3.36 | 1.02 to 5.70 | 2.87 | 66 | 0.006 |
| Selected areas of Honduras | Third follow-up (2022) | Non-modern methods | 1051 | 9.07 | 8.41 | -0.66 | -2.71 to 1.39 | -0.64 | 66 | 0.522 |
| Selected areas of Honduras | Third follow-up (2022) | No response | 1051 | 2.64 | 2.94 | 0.31 | -0.82 to 1.43 | 0.54 | 66 | 0.589 |
| Selected areas of Chiapas, Mexico | Baseline (2012–2013) | No method | 3864 | 57.57 | 44.80 | -12.78 | -14.61 to -10.95 | -13.78 | 180 | <0.001 |
| Selected areas of Chiapas, Mexico | Baseline (2012–2013) | Non-implant modern methods | 3864 | 37.32 | 46.83 | 9.51 | 7.81 to 11.22 | 11.01 | 180 | <0.001 |
| Selected areas of Chiapas, Mexico | Baseline (2012–2013) | Implant | 3864 | 1.26 | 2.13 | 0.88 | 0.02 to 1.73 | 2.03 | 180 | 0.044 |
| Selected areas of Chiapas, Mexico | Baseline (2012–2013) | Non-modern methods | 3864 | 3.25 | 5.73 | 2.48 | 1.75 to 3.22 | 6.70 | 180 | <0.001 |
| Selected areas of Chiapas, Mexico | Baseline (2012–2013) | No response | 3864 | 0.60 | 0.50 | -0.10 | -0.20 to 0.00 | -1.96 | 180 | 0.052 |
| Selected areas of Chiapas, Mexico | Second follow-up (2018) | No method | 1690 | 55.30 | 45.22 | -10.09 | -11.95 to -8.22 | -10.78 | 80 | <0.001 |
| Selected areas of Chiapas, Mexico | Second follow-up (2018) | Non-implant modern methods | 1690 | 37.66 | 44.34 | 6.68 | 4.76 to 8.60 | 6.93 | 80 | <0.001 |
| Selected areas of Chiapas, Mexico | Second follow-up (2018) | Implant | 1690 | 4.31 | 6.79 | 2.48 | 1.44 to 3.52 | 4.75 | 80 | <0.001 |
| Selected areas of Chiapas, Mexico | Second follow-up (2018) | Non-modern methods | 1690 | 1.05 | 1.97 | 0.91 | 0.23 to 1.60 | 2.65 | 80 | 0.010 |
| Selected areas of Chiapas, Mexico | Second follow-up (2018) | No response | 1690 | 1.67 | 1.68 | 0.01 | -0.09 to 0.12 | 0.20 | 80 | 0.845 |

Start and end denote the earliest and latest months of the 24-month retrospective period within each survey. n is the unweighted number of women with both endpoints observed; start and end percentages in this table use this same paired sample. Percentages and contrasts incorporate WEIGHT_WOMAN weights and WTSEG clusters as specified in the analysis code. Differences are end minus start in percentage points (pp). Paired Wald t tests use the joint covariance of the two indicators; degrees of freedom are the number of sampled clusters minus one in this one-stage design. Confidence intervals use the t distribution. P values are two-sided and unadjusted for multiple comparisons; these exploratory comparisons are not comparisons between survey rounds. No response is retained as a category when explicitly coded; missing endpoint values are excluded. These paired estimates may differ from the marginal estimates in Table 1.
